# Genomic epidemiology of a *Cryptococcus neoformans* case cluster in Glasgow, Scotland, 2018

**DOI:** 10.1099/mgen.0.000537

**Published:** 2021-02-23

**Authors:** Rhys A. Farrer, Andrew M. Borman, Teresa Inkster, Matthew C. Fisher, Elizabeth M. Johnson, Christina A. Cuomo

**Affiliations:** ^1^​ Medical Research Council Centre for Medical Mycology, University of Exeter, Exeter EX4 4PY, UK; ^2^​ Public Health England National Mycology Reference Laboratory, Science Quarter, Southmead Hospital, Bristol BS10 5NB, UK; ^3^​ Department of Microbiology, Queen Elizabeth University Hospital, Glasgow, UK; ^4^​ Medical Research Council Centre for Global Infectious Disease Analysis, Imperial College London, London, UK; ^5^​ Broad Institute of MIT and Harvard, Cambridge, MA, USA

**Keywords:** *Cryptococcus neoformans*, Glasgow, infection cluster, mycosis, United Kingdom, whole-genome sequencing

## Abstract

In 2018, a cluster of two cases of cryptococcosis occurred at the Queen Elizabeth University Hospital (QEUH) in Glasgow, Scotland (UK). It was postulated that these cases may have been linked to pigeon droppings found on the hospital site, given there have been previous reports of *Cryptococcus neoformans* associated with pigeon guano. Although some samples of pigeon guano taken from the site yielded culturable yeast from genera related to *Cryptococcus*, they have since been classified as *Naganishia* or *Papiliotrema* spp., and no isolates of *C. neoformans* were recovered from either the guano or subsequent widespread air sampling. In an attempt to further elucidate any possible shared source of the clinical isolates, we used whole-genome sequencing and phylogenetic analysis to examine the relationship of the two *Cryptococcus* isolates from the QEUH cases, along with two isolates from sporadic cases treated at a different Glasgow hospital earlier in 2018. Our work demonstrated that these four clinical isolates were not clonally related; while all isolates were from the VNI global lineage and of the same mating type (MATα), the genotypes of the two QEUH isolates were separated by 1885 base changes and belonged to different sub-lineages, recently described as the intercontinental sub-clades VNIa-93 and VNIa-5. In contrast, one of the two sporadic 2018 clinical isolates was determined to belong to the VNIb sub-lineage and the other classified as a VNIV/VNI hybrid. Our work demonstrated that the two 2018 QEUH isolates and the two prior *C. neoformans* clinical isolates were all genetically distinct. It was not possible to determine whether the QEUH genotypes stemmed from independent sources or from the same source, i.e. pigeons carrying different genotypes, but it should be noted that whilst members of allied genera within the *Tremellomycetes* were isolated from the hospital environment, there were no environmental isolations of *C. neoformans*.

## Data Summary

FASTQ sequences were deposited in the NCBI Short Read Archive under the BioProject accession number PRJNA597039 (https://www.ncbi.nlm.nih.gov/bioproject/?term=PRJNA597039).

**Outcome**

*Cryptococcus neoformans* is a ubiquitous environmental fungus and a major cause of illness in people with compromised immune systems. Genomic studies of *C. neoformans* have demonstrated a huge phylogenetic diversity within the species complex, including sporadic hybrid isolates stemming from two different lineages in both clinical and environmental settings. The high resolution offered by whole-genome sequencing allows the source and cause of clusters of infections to be better understood, and by tracing infection sources may suggest potential remedial measures. In 2018, two cases of cryptococcosis occurred at the Queen Elizabeth University Hospital in Glasgow, Scotland (UK). It was postulated these cases may have been linked to pigeon droppings found on the hospital site, as there have been previous reports of *C. neoformans* associated with pigeon guano. Whole-genome sequencing and phylogenetic analysis of these isolates and two other sporadic infections treated at a different Glasgow hospital earlier in 2018 revealed that none of those isolates from Glasgow were clonally related, as they belonged to well separated sub-clades. Our work therefore suggests that the patients were independently infected. Increased sampling from both hospitals and the environment is necessary to search more precisely for genotypes, and thereby reveal the environmental sources that present the greatest risk for future nosocomial infections.

## Introduction

*Cryptococcus* species are present in a range of environments, especially soil and in association with trees and decaying wood. A number of *Cryptococcus* species, most notably *Cryptococcus neoformans* and *Cryptococcus gattii*, also cause opportunistic infections of humans [1]. *C. neoformans* and related species belong to a large group of *Tremellomycetes* that are commonly associated with bird droppings. For example, the two rarely isolated species *Cryptococcus uniguttulatus* (*Filobasidium uniguttulatum*) and the related species *Papiliotrema laurentii* (previously *Cryptococcus laurentii*) have both been isolated from droppings and cloacal swabs of feral pigeons (*Columba livia*) in Malmö, Sweden, but rarely cause human infection [2]. In contrast, *C. neoformans* is a ubiquitous environmental fungus and a major cause of illness in people living with human immunodeficiency virus (HIV)/AIDS, with an estimated 220 000 cases of cryptococcal meningitis occurring worldwide each year [3].

*C. neoformans* can colonize the intestines of avian species including pigeons and is commonly found in pigeon guano, a natural selective medium that supports mating, unlike its closest relative *C. gattii* [4]. *C. neoformans* is found in both urban and suburban areas, and has been found in up to 8.1 % of samples of pigeon droppings in Iran (*n*=11/136) [5], and up to 15 % pigeon dropping samples (*n*=30) from various provinces in the Nile Delta in Lower Egypt [6]. Proliferation of *C. neoformans* will occur in pigeon guano, particularly in environments protected from sunlight, such as in lofts [7]. Epidemiological and genetic data support transmission of *C. neoformans* from environmental sources including pigeon guano to humans. Infections originate by inhalation of spores directly into the lungs and can be asymptomatic, with some latent cases persisting for months or years before reactivation [8–10].

In 2018, two patients were diagnosed with *C. neoformans* infections at the Queen Elizabeth University Hospital (QEUH) in Glasgow, Scotland (UK). Due to the epidemiological linkage of these cases, an investigation was initiated to search for a common source, including pigeon guano found on the hospital site. In this study, we employed whole-genome sequencing and phylogenetic analysis to understand the pathogen genetics underpinning these cases.

## Methods

### Clinical case summaries

Infections were diagnosed based on blood cultures that were positive for *C. neoformans*, following episodes of pyrexia. This prompted referral to the infection control team for investigation. The 73-year-old female patient was undergoing treatment for relapsed T cell lymphoma and the 10-year-old male had recently completed treatment for Burkitt’s lymphoma. Both patients had received fluconazole prophylaxis during their hospital stay; however, due to development of side effects or interactions, both patients had a time period with a gap in cryptococcal cover, where the antifungal was either discontinued or they were switched to another agent. This was a risk factor for cryptococcal acquisition or reactivation.

### Epidemiological investigation

Due to the epidemiological link in time and place, further investigations by the infection control team and estates colleagues were initiated, and revealed evidence of pigeons and pigeon guano in the plant room on the top floor of the hospital and in various external areas, such as window ledges and courtyards. Limited environmental sampling was undertaken to search for a source of the infections and before implementation of control measures. Following cleaning and pest-reduction measures on site, no further cases of cryptococcal infection were identified. Numerous hypotheses were investigated; however, the exact route of transmission has not been elucidated. There were pitfalls to the initial environmental sampling. Following the finding of pigeon guano, superficial swabs were taken from areas of contamination within the plant room. This was not deemed a sufficient quantity of material by the veterinary laboratory, which requested further pigeon guano sampling in the form of several pots or specimen containers. At the time of the repeat sampling, the plant room had been cleaned; pigeon guano was retrieved from another area of the campus, with no detection of *C. neoformans*. Similarly, with regard to air sampling, initial agar plates were incubated for 5 to 7 days and were overgrown with other environmental fungi such as *Aspergillus*. However, ideally these plates should have been read for *Cryptococcus* from 48 h with the use of a selective agar. Subsequent air sampling was again undertaken after the plant room had been cleaned.

### *Cryptococcus* isolation, genomic DNA isolation and DNA sequencing

The four isolates of *C. neoformans* analysed here were recovered from blood cultures from four independent patients, the two patients from the cluster of cases at QEUH and two unrelated patients from independent hospitals in the same city (Glasgow). Isolate identity was confirmed at the UK National Mycology Reference Laboratory by MALDI-TOF MS analyses, as described elsewhere [9], after confirmation of isolate purity by culture on CHROMagar *Candida* chromogenic media. Fungal genomic DNA for whole-genome sequencing was extracted from 4-day-old cultures grown on Sabouraud agar. *C. neoformans* cells were harvested by scraping into sterile water, subjected to bead-beating for 2×30s at 6.5 m/s in a FastPrep-24 instrument (MP Biomedicals) and liberated DNA was purified using Qiagen MiniBlood extraction columns, according to the manufacturer’s instructions. Genomic DNA extracted from approximately 5×10^9^ cells was eluted in 200 µl final volumes of sterile water and the DNA concentration was measured using Qubit dsDNA HS reagent. Libraries were constructed using the Illumina Nextera Flex protocol and sequenced on an iSeq 100 to generate paired 150 bp reads. Total sequence coverage of the H99 reference genome [11] ranged from 37× to 54× depth.

### Genome alignment, SNP identification and phylogenetic analysis

Illumina reads were aligned to the *C. neoformans* var. *grubii* H99 genome version CNA2 [11]. For the hybrid isolate 18E21177, reads were aligned to both H99 and the VNIV genome JEC21 isolate [11] using bwa-mem v0.7.4 [12] with default parameters and converted to sorted BAM format using SAMtools v1.8 [13]. Variants were called using the same tools and parameters used by Desjardins *et al.* to analyse 387 *C. neoformans* genomes [14]. Briefly, variants were identified using Genome Analysis Tool Kit (GATK) version 3.4 [15]. First, indels were locally realigned, HaplotypeCaller was invoked in GVCF mode with ploidy=1, and genotypeGVCFs was used to predict variants in each isolate. All variant call format (VCF) files were then combined and sites were filtered using VariantFiltration with QD <2.0, FS >60.0 and MQ <40.0. Individual genotypes were then filtered if the minimum genotype quality was <50, per cent alternate allele <0.8 or depth <10.

VCF files from the three newly sequenced isolates were merged with the 387 isolates analysed by Desjardins *et al.* [14] using VCFtools vcf-merge [16]. The multi-sample VCF file was converted to FASTA with a ECATools (https://github.com/rhysf/ECATools) with the following criteria: (i) ignore any non-homozygous or non-single base allele for a given isolate, (ii) exclude sites that were >10 % ambiguous amongst the 390 isolates, and (iii) use an ‘N’ for any site that was ambiguous but not excluded, resulting in 1 196 579 sites per isolate. A phylogenetic tree was reconstructed using FastTree v2.1.3 SSE3 [17]. Principle component analysis was performed using SmartPCA v4 [18]. Evidence for aneuploidy or copy number variation (CNV) was assessed using non-overlapping 10 kb windows of normalized depth of coverage plots.

## Results

### Case description and epidemiological evaluation

In 2018, two patients were diagnosed with *C. neoformans* infections at the QEUH in Glasgow, Scotland. These infections were identified over a 17 day period in late 2018. Both patients (one adult and one child) had underlying haematological malignancies. Prior to blood culture testing, both patients had been in hospital for a prolonged period of time (the child for several months and the adult patient for 15 days). Both patients were from the UK, and there was no recent history of overseas travel, contact with birds or occupational risk factors. A single case of *C. neoformans* in an adult patient may ordinarily have been deemed sporadic and likely representative of reactivation. However, the development of a second case 17 days later in a child who had not left hospital for over 3 months led to the consideration of whether the *C. neoformans* infections were hospital acquired from a potential source on the site. Pigeon droppings contained culturable yeast from genera related to *Cryptococcus* but have since been classified as *Naganishia* or *Papiliotrema* species, and some sources linked this finding with the patient cases [19].

### Genomic analysis of QEUH isolates

**Fig. 1.**
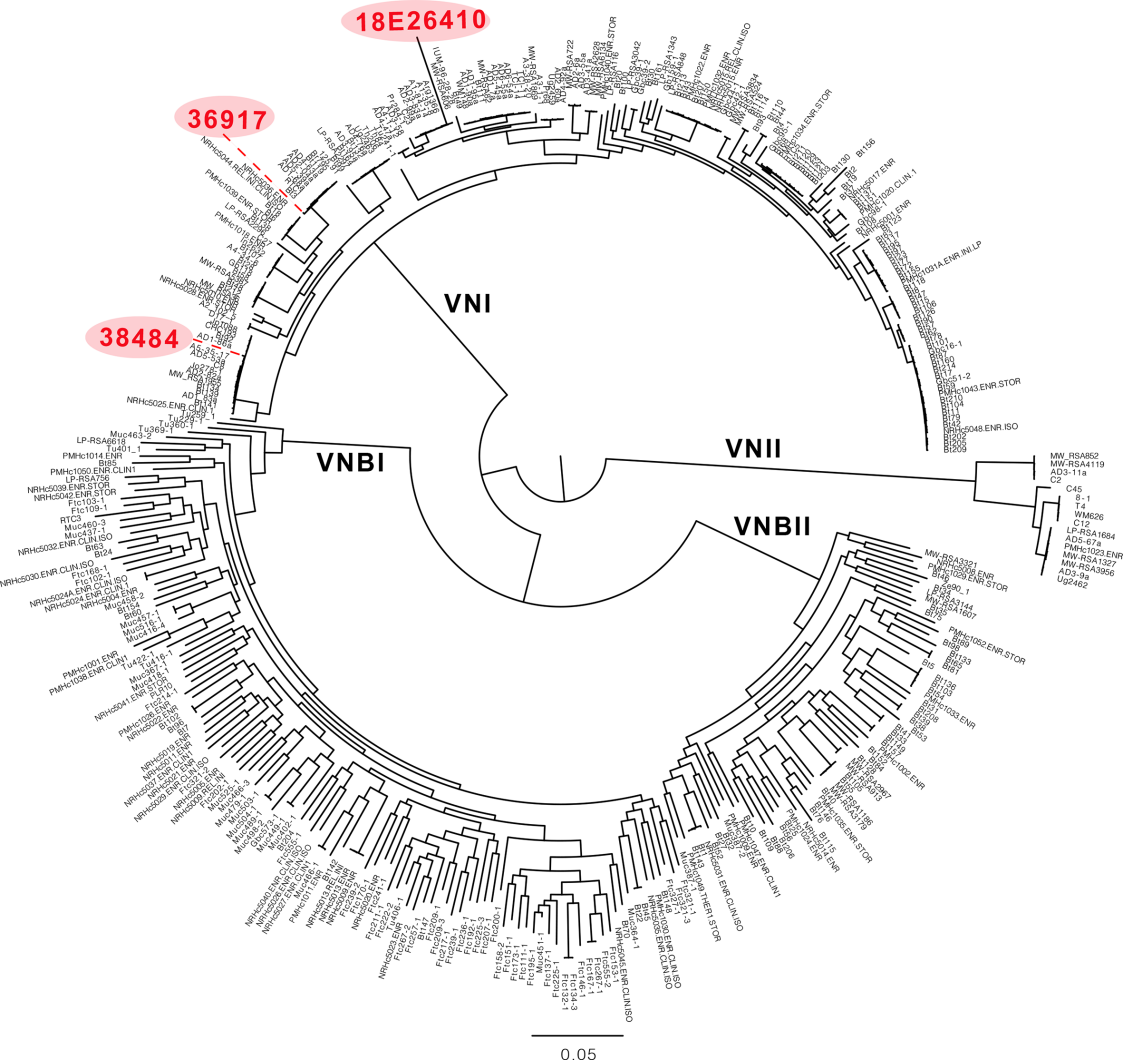
FastTree based on the isolates from the Desjardins *et al.* paper [14], along with the three non-hybrid isolates from the QEUH study highlighted in red. Branch lengths/scale bar show the number of substitutions per site.

**Fig. 2.**
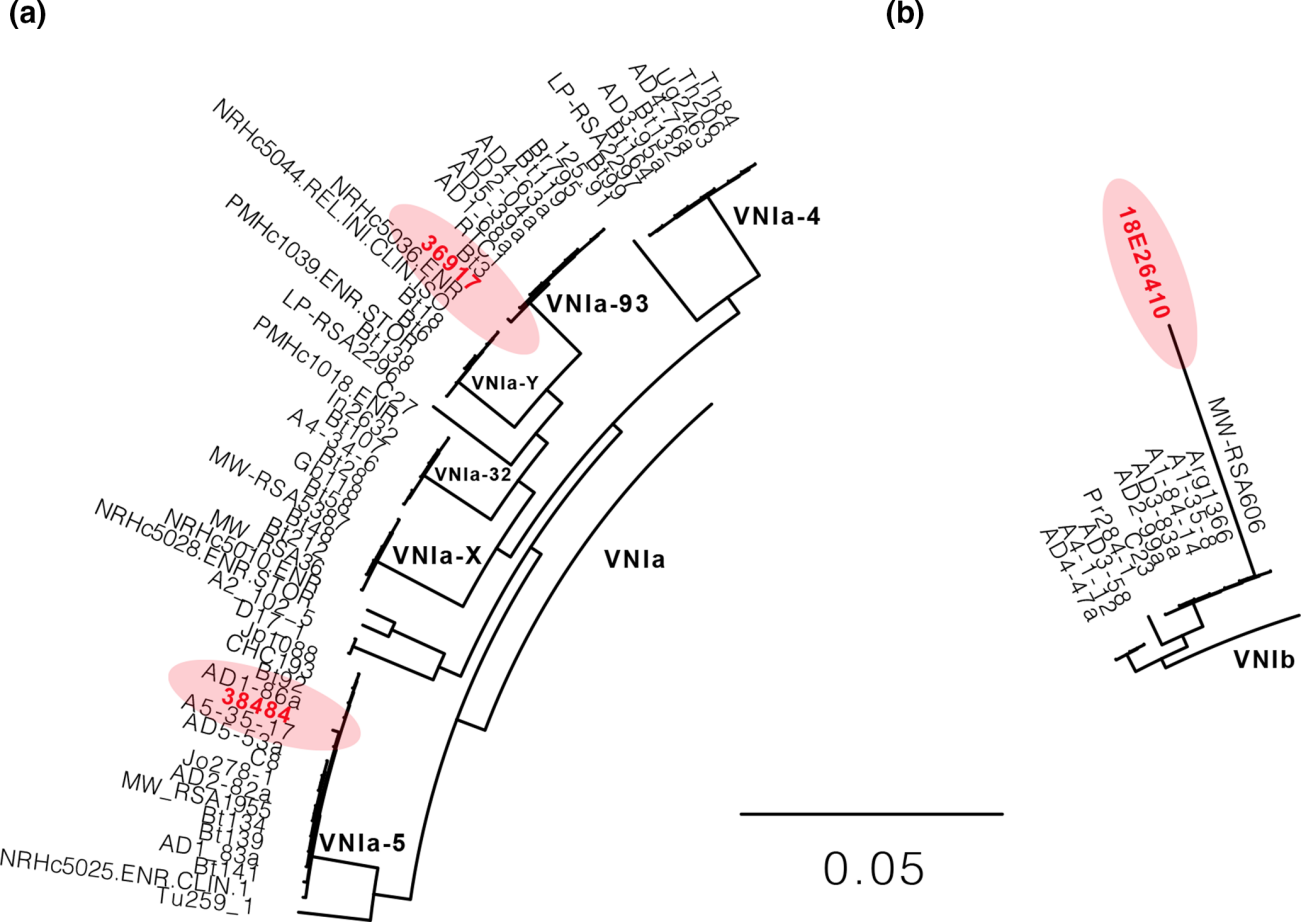
A subsection of Fig. 1 showing the relationships of the non-hybrid isolates 38484, 36917 (a) and 18E26410 (b) from the QEUH study highlighted in red, compared with other sub-clades presented in the Desjardins *et al.* paper [14]. Branch lengths/scale bar show the number of substitutions per site.

**Fig. 3.**
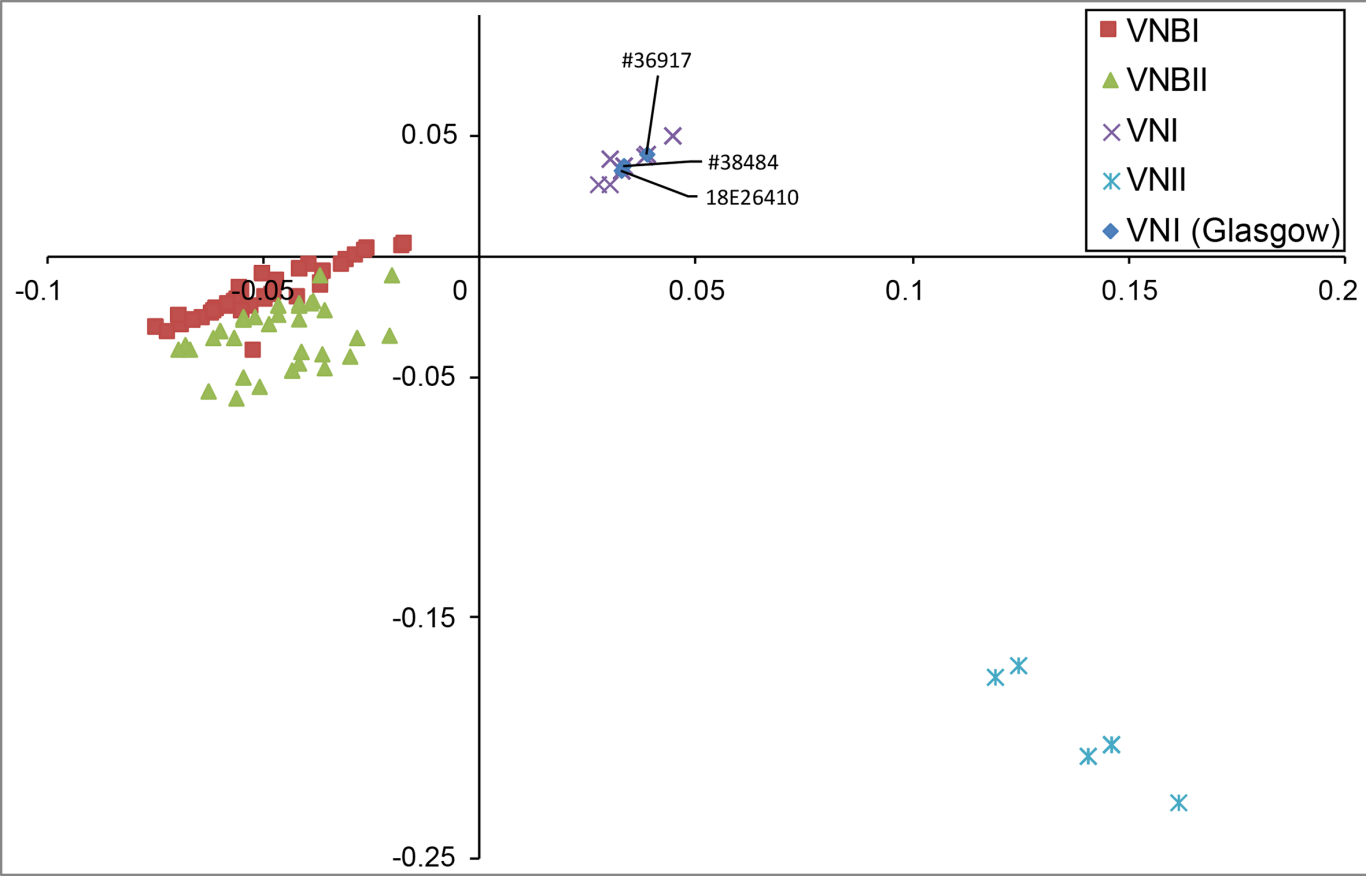
Principle component analysis based on SmartPCA. All isolates from the QEUH cluster together with other VNI clade isolates.

Sequence and phylogenetic analysis revealed that the two isolates from the 2018 QEUH cases were not clonally related. By identifying variants and comparison to a set of 387 diverse *C. neoformans* isolates [14], phylogenetic analysis demonstrated that the two QEUH isolates belong to the global VNI lineage of *C. neoformans* var*. grubii* (serotype A); they fall within two different sub-clades of the VNIa sub-lineage (Figs 1–3). Two additional isolates from sporadic infections earlier in 2018, from independent Glasgow hospitals, displayed even higher genetic divergence; one (18E26410) belonged to the VNIb clade, while a fourth isolate (18E21177) was a *C. neoformans* var*. grubii* (serotype A)/*C. neoformans* var*. neoformans* (serotype D) hybrid and, thus, excluded from phylogenetic analysis.

To determine how genetically distinct the two QEUH *C. neoformans* isolates were (thereby determining how genetically distinct the VNIa-93 sub-clade is from VNIa-5), we compared variant sites between the two case isolates 36917 and 38484 (see below), which identified 1885 genetic differences, most of which were found in intergenic and intronic regions. Genetic differences mapped to predicted H99 transcripts revealed 455 changes between the two clades, including 166 single base changes, 88 of which caused non-synonymous changes in coding sequences. No readthrough or nonsense mutations were detected between the two sub-clades, although 23 differences involved frameshifts. Thus, the two sub-clades are slightly different at both the genetic and predicted transcript level.

The first 2018 isolate from QEUH (isolate 36917) belongs to the VNIa-93 sub-clade, based on the assignment of other isolates that fell within the same sub-clade, including isolate RTC1 from Ashton *et al.* [20], and RTC1 and Bt3 from Desjardins *et al.* [14]. Both RTC1 and Bt3 were collected from HIV^+^ patients in Botswana [21, 22]. The VNIa-93 sub-clade is the most common sub-clade found in Uganda and Malawi, but is also common in Vietnam (12 %, *n*=44/762) and found more rarely elsewhere in the world, including Botswana, Laos, France and Brazil [20]. Indeed, this lineage accounted for 20 % of all isolates in the Ashton *et al.* study, and was suggested to be one of the three sub-clades responsible for recent population expansion of *C. neoformans* [20]. VNIa-93 was also associated with better outcomes than other major global *C. neoformans* sub-clades VNIa-4 and VNIa-5, which predominate in South-East Asia [20].

The second 2018 isolate from QEUH (isolate 38484) belongs to the VNIa-5 sub-clade, based on the assignment of other isolates that fell within the same clade, including AD1-86a in both Ashton *et al.* [20] and Desjardins *et al.* [14]. VNIa-5 is the second of the three sub-clades responsible for the recent population expansion of *C. neoformans* [20]. VNIa-5 predominates in South-East Asia, but has also been reported in a few patients in Africa, USA, China and Japan [20]. VNIa-5 accounted for 21 % isolates (*n*=163/762) analysed in the Ashton *et al.* study [20]. Importantly, we confirmed that the VNIa-93 isolate 36917 and the VNIa-5 isolate 38484 are genetically distinct, and represent discrete ancestral histories, and that the cases at QEUH in Glasgow, UK, were caused by isolates from two separate sub-clades.

Based on the sequence analysis, both QEUH isolates were haploid and lacked any evidence for aneuploidy (Fig. S1, available with the online version of this article). Our analysis also demonstrated little evidence of CNV. Both aneuploidy and CNV have been previously observed in *C. neoformans* and can contribute to drug resistance [23, 24].

Two further isolates were identified from another hospital in the same city in 2018. 18E26410 was a blood culture isolate from a 72-year-old male and 18E21177 was a blood culture isolate from a 68-year-old male. These cases were determined to be sporadic as they were not linked in time, place or person. Neither patient had a history of a significant hospital stay prior to their positive result, although one patient spent 48 h in the same hospital as the cluster under investigation, in April 2018. Isolate 18E26410 belonged to VNIb and was included in the phylogenetic analysis (Figs 1–3). Based on phylogenetic and genome analysis, isolate 18E21177 is a *C. neoformans* var. *neoformans* (VNIV)/*C. neoformans* var*. grubii* (VNI) hybrid, and was subsequently excluded from the phylogenetic analysis (Figs S2 and S3).

## Discussion

In this study, we used whole-genome sequencing to investigate the relationship between a cluster of two contemporaneous and hospital-linked cases of *C. neoformans,* two additional local isolates, as well as a globally collected data set. Both QEUH isolates were assigned to the VNIa global sub-lineage although they belong to different clades. The large number of variants that differentiate the two isolates from 2018 supports a model of independent acquisition by each patient, rather than transmission of a clonal isolate.

Only one other cluster of *C. neoformans* infections in hospitalized patients has been reported in the literature. In Arkansas in 2013, six patients in a community hospital developed bloodstream and respiratory infections. Bird habitats at the hospital and staff who had contact with birds were investigated, but no definitive source was established, and environmental sampling was negative. Isolates from these clinical cases appeared genetically diverse, as three separate MLST (multilocus sequence typing) types were identified [25].

In the 2018 Glasgow incident, it is possible that patients acquired *C. neoformans* from plant-room contamination entering the ventilation system or voids, or from ingress of spores into the building from external air. Alternatively, cryptococcal reactivation or recent infection prior to hospitalization is a possibility, but would seem less likely in the context of epidemiological links in time, place and person with a feasible source.

If whole-genome comparisons had revealed that the two QEUH isolates were highly genetically similar, this would have strengthened the argument that they arose from a point source and, thus, were likely to be linked to a single nosocomial source. However, the fact that they are genetically distinct does not necessarily rule out a common source of infection, given that pigeon guano from different birds, and even from the same bird, may contain a variety of unrelated genotypes due to the general diversity of environmental isolates. Although other *Tremellomycetes* yeasts were found in the locality, there was no environmental isolation of *C. neoformans* from the hospital buildings or from the wide-scale air sampling undertaken following the identification of the second case, which would have added an extra dimension to the study.

While an epidemiological link in time and place to a pigeon infestation and guano detection on the hospital site suggested a common source, genome sequencing of the two cryptococcal isolates involved did not provide evidence of a single genotype causing infection. However, there were several limitations in the environmental sampling, including both culturing conditions and the cleaning of potential source sites, which may have decreased the likelihood of detecting *C. neoformans* in subsequent samples. Therefore, we cannot exclude a point source consisting of multiple cryptococcal strains. Indeed, genetic diversity of clinical and environmental isolates within a city has been described [26]. While wider sampling might reveal some isolates with closer genetic links, this study, including isolates from two other infections from the same geographical area, highlights the diversity of genotypes causing infection in the UK.

## Supplementary Data

Supplementary material 1
