# Supplementary material for: Genomic epidemiology of a Cryptococcus neoformans case cluster in Glasgow, Scotland, 2018
Source: Microb Genom. 2021 Feb 23;7(3):mgen000537. doi: 10.1099/mgen.0.000537 (PMC8190611; doi:10.1099/mgen.0.000537)
Supplement: Supplementary material 1 [file mgen-7-0537-s001.pdf]

Supplementary Material

Genomic epidemiology of a *Cryptococcus neoformans* case cluster in Glasgow, Scotland, 2018.

Rhys A. Farrer[1], Andrew M. Borman[1,2], Teresa Inkster[3], Matthew C. Fisher[4], Elizabeth M. Johnson[1,2], Christina A. Cuomo[5]

[1]Medical Research Council Centre for Medical Mycology, University of Exeter, Exeter, EX4 4PY, United Kingdom
[2]Public Health England National Mycology Reference Laboratory, Science Quarter, Southmead Hospital, Bristol, United Kingdom BS10 5NB
[3]Department of Microbiology, Queen Elizabeth University Hospital, Glasgow, Scotland
[4]MRC Centre for Global Infectious Disease Analysis, Imperial College London, London, United Kingdom
[5]Broad Institute of MIT and Harvard, Cambridge, Massachusetts, United States of America

Corresponding authors: Rhys A. Farrer (r.farrer@exeter.ac.uk), Christina A. Cuomo (cuomo@broadinstitute.org)

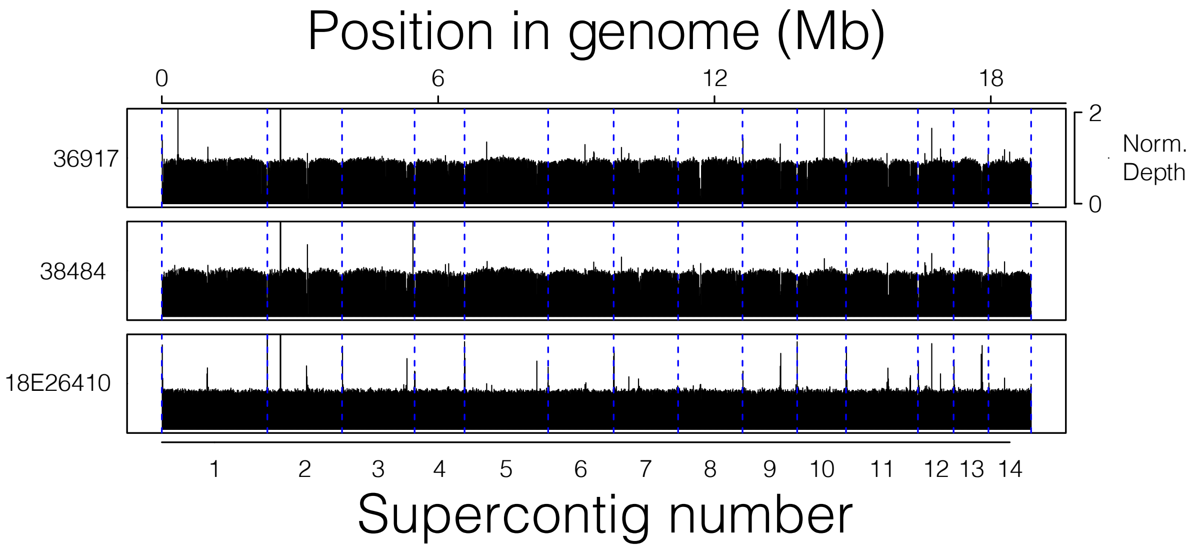

Supplementary Figure1. Normalized depth of coverage (depth of coverage / depth of sequencing across genome) was calculated across 10 kb non-overlapping windows for the three non-hybrid isolates.

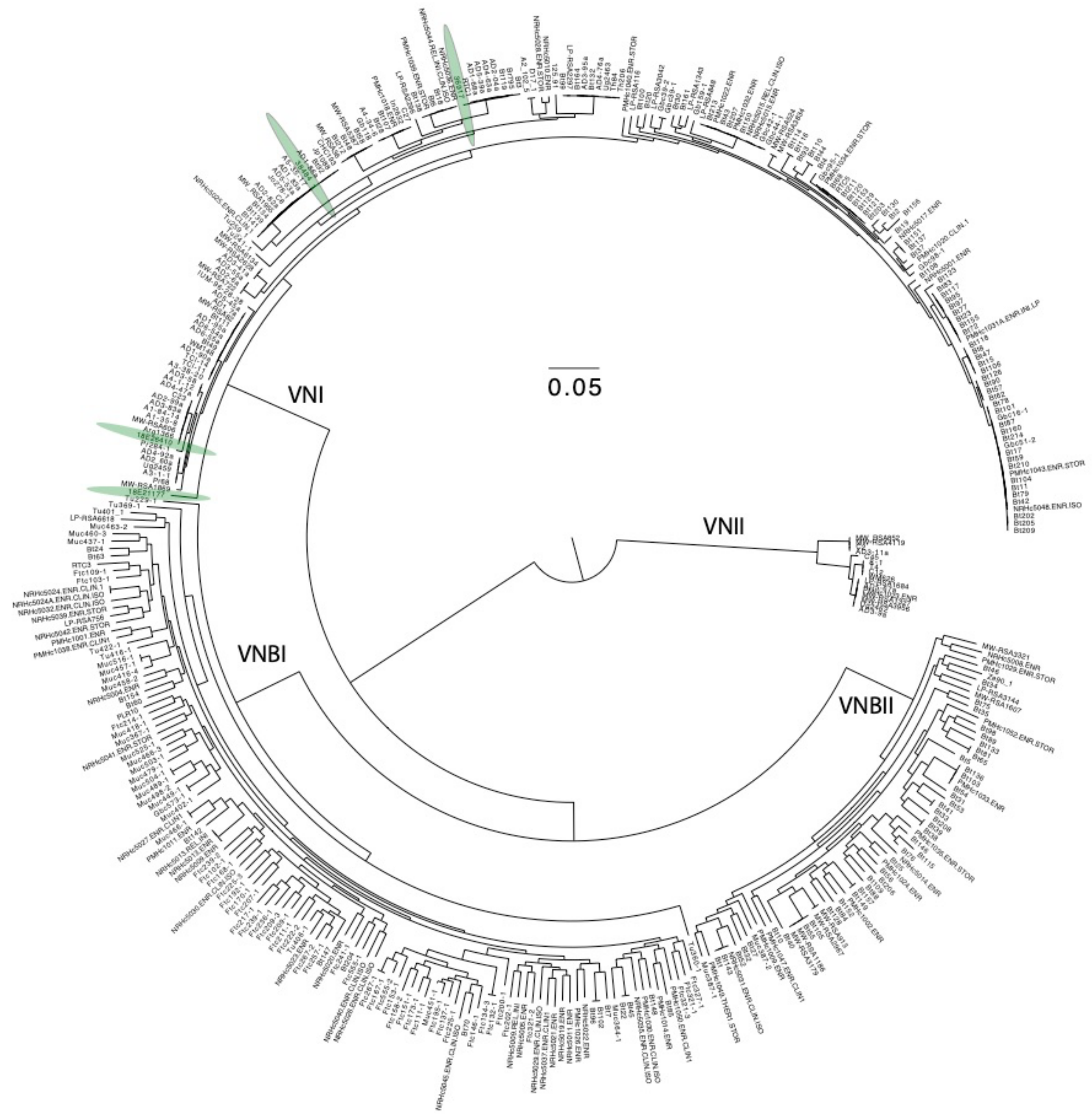

Supplementary Figure2. FastTree [1] based on the isolates from the Desjardins *et al.* paper [2], along with all four isolates (including the hybrid 18E21177) from this study highlighted in green.

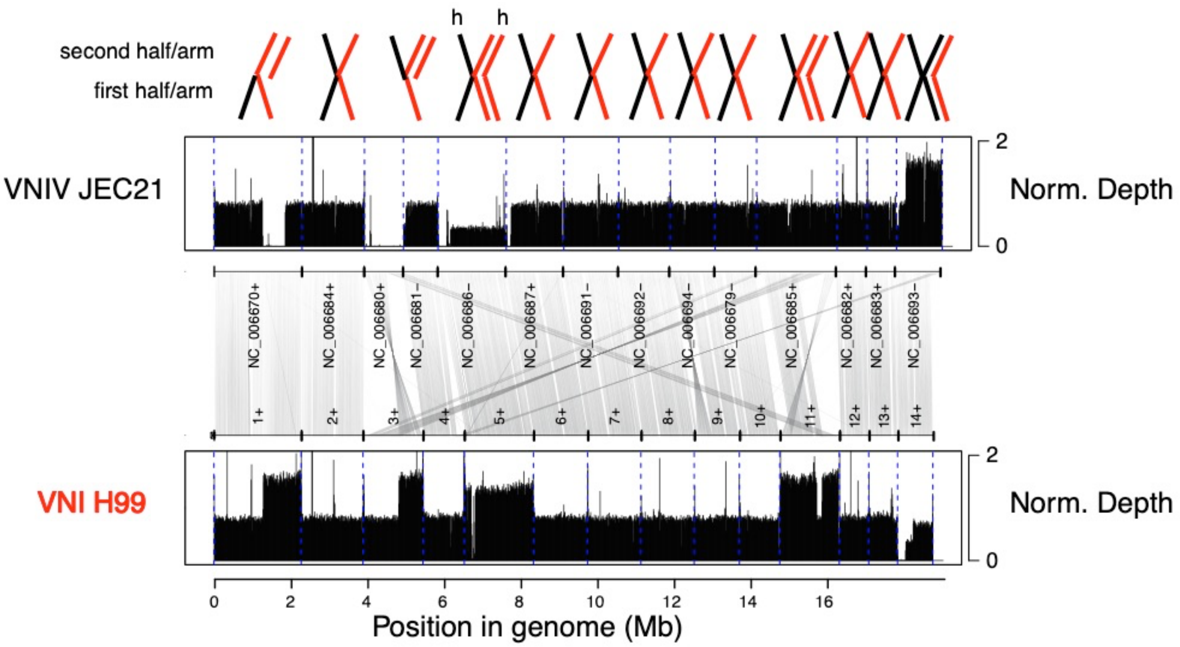

Supplementary Figure3. 18E21177 sequence reads were aligned to both the VNI H99 genome (*C. neoformans* var. *grubii*, serotype A) (red) and the VNIV JEC21 genome (*C. neoformans* var. *neoformans*, serotype D) (black). Normalized depth of coverage for non-overlapping windows was calculated and plotted, demonstrating aneuploidy across both parental genotypes (H99 or JEC21). Synima [3] was used to identify orthologs between H99 and JEC21 and reveal synteny between the assemblies. The depth of coverage plots are ordered by chromosome: H99 from Chr1 to Chr14, which corresponds to a non-linear order of contigs in JEC21 (E.g. Chr1 = NC_006670, Chr2 = NC_006884, Chr3 = NC_006680) etc. The top of the plot summarizes the lineage ancestry for 18E21177, which is mostly diploid, with largely a copy of each chromosome from both parental lineages, although there are some examples of potential hemi-zygosity (labelled with an 'h' on the figure) and disomy. With the exception of H99 Chr11, all aneuploid H99 chromosomes appear to have corresponding ploidy changes in the JEC21 chromosomes, potentially indicating chromosome loss and reduplication during parasex. Due to the placement of 18E21177 in the tree (Sup. Fig. 2) and this evidence of hybridization, this isolate was excluded from further phylogenetic work, including sub-clade placement.

References

1. **Price MN, Dehal PS, Arkin AP**. FastTree: computing large minimum evolution trees with profiles instead of a distance matrix. *Mol Biol Evol* 2009;26:1641–1650.

2. **Desjardins CA, Giamberardino C, Sykes SM, Yu C-H, Tenor JL, *et al.*** Population genomics and the evolution of virulence in the fungal pathogen *Cryptococcus neoformans*. *Genome Res* 2017;27:1207–1219.

3. **Farrer RA**. Synima: a synteny imaging tool for annotated genome assemblies. *BMC Bioinformatics* 2017;18:507.
